# Structural Aspects of Drug Resistance and Inhibition of HIV-1 Reverse Transcriptase

**DOI:** 10.3390/v2020606

**Published:** 2010-02-11

**Authors:** Kamalendra Singh, Bruno Marchand, Karen A. Kirby, Eleftherios Michailidis, Stefan G. Sarafianos

**Affiliations:** Christopher Bond Life Sciences Center & Department of Molecular Microbiology & Immunology, University of Missouri, Columbia, Missouri 65211, USA; E-Mails: singhka@missouri.edu (K.S.); marchandb@missouri.edu (B.M.); kirbyk@missouri.edu (K.A.K.); em6f4@mail.missouri.edu (E.M.)

**Keywords:** HIV-1 reverse transcriptase, nucleoside RT inhibitors, non-nucleoside RT inhibitors, translocation defective RT inhibitors, drug resistance, excision

## Abstract

HIV-1 Reverse Transcriptase (HIV-1 RT) has been the target of numerous approved anti-AIDS drugs that are key components of Highly Active Anti-Retroviral Therapies (HAART). It remains the target of extensive structural studies that continue unabated for almost twenty years. The crystal structures of wild-type or drug-resistant mutant HIV RTs in the unliganded form or in complex with substrates and/or drugs have offered valuable glimpses into the enzyme’s folding and its interactions with DNA and dNTP substrates, as well as with nucleos(t)ide reverse transcriptase inhibitor (NRTI) and non-nucleoside reverse transcriptase inhibitor (NNRTIs) drugs. These studies have been used to interpret a large body of biochemical results and have paved the way for innovative biochemical experiments designed to elucidate the mechanisms of catalysis and drug inhibition of polymerase and RNase H functions of RT. In turn, the combined use of structural biology and biochemical approaches has led to the discovery of novel mechanisms of drug resistance and has contributed to the design of new drugs with improved potency and ability to suppress multi-drug resistant strains.

## 1. Introduction

The reverse transcription of the viral single-stranded (+) RNA genome into double-stranded DNA (dsDNA) is an essential step in the replication of HIV. While several viral proteins and enzymes participate in the process of conversion of RNA to DNA, the reverse transcription is entirely dependent on the activities of an enzyme called reverse transcriptase (RT) [1]. The human immunodeficiency virus type I reverse transcriptase (HIV-1 RT) has two distinct activities: (i) a DNA polymerase activity, which uses either RNA or DNA as template and (ii) an RNase H activity, which degrades RNA from RNA/DNA hybrids endonucleolytically [2]. Unlike many other DNA polymerases, HIV-1 RT does not have proof-reading activity. Thus, error-prone DNA synthesis carried out by HIV-1 RT results in enhanced mutation frequency and the production of multiple HIV variants.

**Figure 1.**
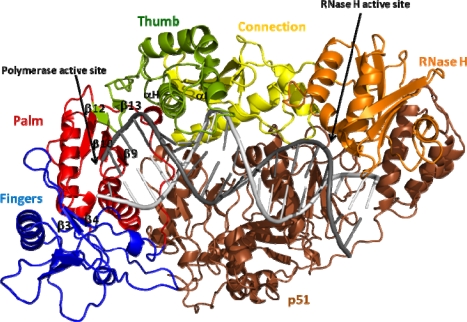
The crystal structure of HIV-1 RT bound to double stranded DNA (PDB code 2hmi). HIV-1 RT functions as heterodimer of p66 and p51 subunits. Due to the resemblance of p66 to a closed right hand, subdomains of p66 have been named as the ‘palm’ (red), fingers (blue), and thumb (green). The p66 subdomain contains two active sites, the polymerase and the RNase H active sites (orange). The region between the RNase H and polymerase active sites is known as the connection (yellow) subdomain. The p51 (dark brown) subunit is derived from the proteolytic cleavage of RNase H from p66 and has identical primary and secondary structure. However, the tertiary structure of p51 is markedly different than p66 leading to a non-functional arrangement of catalytic residues. The template/primer (white/gray) is seen in the DNA-binding cleft formed primarily by the p66 subunit of the enzyme. Figures 1, 2, 3, 7, 9 and 11 were generated using PyMOL [4].

HIV-1 RT is a heterodimer composed of two subunits, a 66 kDa (p66) and a 51 kDa (p51) subunit (Figure 1). The p66 subunit is 560 amino acids long and contains the active sites of the polymerase and RNase H functions of the enzyme; the p51 subunit contains the first 440 amino acids of p66 and is derived from HIV-1 protease-mediated cleavage of the RNase H domain from the p66 subunit [3].

## 2. HIV-1 RT Function in Viral Replication

HIV-1 RT converts the viral RNA into dsDNA in the cytoplasm of the infected cell. The resulting linear double-stranded viral DNA is transported into the nucleus where it is integrated into the host genome by HIV integrase (IN). This integrated DNA copy, called a provirus, is the source of both viral genomic and viral messenger RNAs, which are generated by the host DNA-dependent RNA polymerase. Although other viral proteins (notably the nucleic acid chaperone nucleocapsid and perhaps IN), and probably some cellular factors, help RT to carry out the reactions that convert the viral RNA into DNA, RT contains all the necessary enzymatic activities for such conversion.

Similar to other template-dependent DNA polymerases, RT requires both a primer and a template to initiate DNA replication. After fusion of the HIV particle followed by un-coating of the capsid, the DNA replication by HIV-1 RT is initiated from the host tRNA_lys3_. An 18-nucleotide sequence near the 5′-end of the HIV genome is called the primer binding site (PBS) and is complementary to the 18 nucleotides at the 3′-end of tRNA_lys3_. The viral RNA genome, which acts as the template, is plus-strand. During the first (minus) strand DNA synthesis, RT uses the tRNA_lys3_ as a primer and copies the 5′– end of the viral RNA genome, generating an RNA/DNA hybrid that is a substrate for RNase H. The RNA/DNA hybrid is degraded to generate a nascent minus-strand single-stranded DNA. The sequences at the 5′– and 3′–ends of the viral RNA genome are identical (repeat or R). This allows the minus-strand DNA to hybridize with the R sequence at the 3′-end of one of the two viral RNAs in the virion. This step is called the minus-strand transfer. After the hybridization of nascent DNA to R sequence, the minus-strand DNA synthesis continues using viral RNA as a template. As DNA synthesis proceeds, RNase H degrades the RNA strand. Although most of the RNase H cleavages are not sequence specific, a specific sequence rich in purines (called the polypurine tract or PPT) near the 3′-end of the viral RNA is relatively resistant to cleavage by RNase H. Therefore, it serves as the primer for second (plus) strand DNA synthesis. Plus-strand DNA synthesis proceeds and is followed by the removal of the tRNA primer by RNase H. This leads to the second (plus-strand) transfer, which is followed by extension of both the minus and plus strands until the entire DNA is double stranded, creating a DNA that has the same sequences at both ends (these repeats are called long terminal repeats or LTRs).

## 3. A Historical Perspective of Three-Dimensional Structures of HIV-1 RT

During the past 18 years, numerous crystal structures of HIV-1 RT have revealed the molecular details of the mechanisms of DNA polymerization, RNase H cleavage, inhibition, and drug resistance [5–17]. In 1992, a low resolution (7 Å) electron density map of an HIV-1 RT/DNA complex revealed that the nucleic acid binds in a groove on the surface of the enzyme and that the polymerase and RNase H active sites are at the opposite sides of this nucleic acid binding cleft [18]. The molecular details of the RT secondary structure and folding were revealed soon after in the 3.5 Å structure of HIV RT in complex with nevirapine, a non-nucleoside reverse transcriptase inhibitor (NNRTI) [17]. In this structure, it was revealed that the p66 and p51 subunits fold differently and that the nucleic acid binding cleft is analogous to that of the Klenow fragment of the *Escherichia coli* DNA pol I structure, which was the only other polymerase structure known at the time [19]. Based on the similarity of the RT and Klenow fragment structures to a half-open right hand [18], various conserved subdomains were designated as fingers (residues 1–85 and 118–155), palm (residues 86–117 and 156–236), and thumb (237–318) subdomains. The region that connects the RT thumb subdomain to the RNase H subdomain (residues 427–560) was called connection subdomain (residues 319–426) [12,17,20]. This anthropomorphic description has been helpful in referring to structural regions of the various RTs and has been adopted in the description of structures of other DNA and RNA polymerases. Nevirapine was bound in a pocket near but not overlapping with the polymerase active site. Soon after, the complex of HIV-1 RT with DNA substrate was solved independently at 3.0 Å. This structure was the first to provide the molecular details of any DNA polymerase interacting with its nucleic acid substrate [20] at the polymerase active site. In this structure (later refined at 2.8 Å [9]), it was shown that the bound template/primer had both A-form and B-form regions separated by a 45° bend. The distance between the polymerase and RNase H active sites was 17–18 DNA base pairs. Most RT-DNA interactions involved the phosphate backbone of the DNA and residues of the palm, thumb, and fingers of p66. The nucleic acid was positioned at the polymerase active site by residues of the p66 palm subdomain together with two alpha-helices of the p66 thumb (αI and αH). The catalytically essential D110, D185, and D186 residues were seen close to the 3′-OH of the primer terminus.

The 3.2 Å structure of unliganded RT [7] revealed a striking difference between RT and both RT/DNA and RT/nevirapine complex structures [17,20]. This difference was the major conformational rotation of the p66 thumb subdomain that accompanied the binding of DNA or NNRTI. These structural changes were confirmed by a higher resolution (2.7 Å) unliganded structure [6]. In a separate study, it was shown that unliganded RT crystals (2.35 Å resolution) prepared by soaking out the NNRTI from pre-grown crystals of an RT/NNRTI complex can assume a different conformation where the p66 thumb subdomain is similar to the parent RT/NNRTI complex [5]. This unusual conformation is likely the result of the method by which these crystals were prepared. The solution of the 2.2 Å structure of HIV-1 RT in complex with nevirapine, was an important development because even if the same structure was previously solved by Steitz and colleagues [17], this structure provided the first high-resolution details of the interactions between RT and nevirapine [21]. Glimpses of the atomic details of the p66 palm and thumb subdomains of RT were provided earlier by the 2.2 Å resolution structure of the N-terminus of RT by Unge *et al*. [22]. Numerous other NNRTI structures helped highlight similarities of NNRTI binding at the NNRTI-binding pocket. Other RT structures in complex with substrates have provided important information regarding the mechanism of RNAse H cleavage, DNA polymerization, and drug resistance. The 3.0 Å structure of the HIV-1 RT complex with RNA/DNA containing the PPT sequence elucidated the molecular interactions between RT and this structural element, offering insights into the initiation of second strand synthesis during reverse transcription [8]. Also, the 3.0 Å structure of the pre-translocation reaction intermediate of polymerization provided structural insights into this elusive step of DNA synthesis [20].

A key landmark was the solution of the 3.2 Å structure of HIV-1 RT in complex with DNA and dTTP [12]. This structure revealed that binding of dNTP is accompanied by important conformational changes of the p66 fingers subdomain. It also provided glimpses of interactions that are responsible for drug resistance. Additional information on the interactions of HIV-1 RT active site residues with NRTI or dATP came from the structure of wild-type (WT) and drug-resistant HIV RT (K65R) in complex with DNA and tenofovir diphosphate or dATP [23]. Throughout the years, structural studies with HIV-1 RT mutant enzymes in complex with substrates or NNRTIs have helped determine the mechanisms of DNA polymerization and drug resistance [5,8,10–14,16–17,20–21,23–30].

## 4. Structural Features of HIV-1 RT

### 4.1. Nucleic Acid Binding Cleft

The nucleic acid binding cleft of HIV-1 RT is formed by the fingers, palm, thumb, connection, and RNase H subdomains of the p66 subunit. The p51 thumb and connection subdomains form the floor of the cleft (Figure 1). In all crystal structures of RT in complex with nucleic acids, the DNA/DNA or RNA/DNA contact simultaneously both the polymerase and the RNase H domains of RT, separated by 17–18 base pairs of the nucleic acid substrate. While the 3′-end of the primer strand is always engaged at the polymerase active site, the RNase H active site is not seen to contact the scissile phosphate in any of the crystallized complexes. Nonetheless, biochemical data suggest that it is possible for RT to engage a template/primer at both active sites simultaneously [35].

The interactions of RT with the nucleic acid involve primarily the sugar-phosphate backbone and therefore these interactions are non-specific. Most interactions are between the six primer, and eight template nucleotides proximal to the polymerase active site, with the fingers, palm, and thumb subdomains of p66. Residues of the p66 fingers form the ‘template grip’ (V75, R78, N81, E89, P157, and G93), have close contacts with base-paired residues of the template strand and help position the template at the polymerase active site [8,9], while others (W24, F61) help to bend the unpaired 5′ template overhang away from the helical axis of the nucleic acid duplex by making hydrophobic contacts with nucleotide bases [12,20,23]. Interactions of the nucleic acid with the p66 palm subdomain involve amino acid residues from the polymerase active site region, including M184, which interacts with the 3′-OH primer terminus and is part of the highly conserved YMDD motif present in all retroviral RTs [36–38]. The primer strand interacts with p66 palm domain residues that form the “DNA primer grip”, a highly conserved structural motif close to the polymerase active site [39], which comprises the p66 β12–β13 hairpin in HIV-1 RT and helps position the 3′-OH end of the primer strand at the polymerase active site. In addition, residues of the “RNase H primer grip” in the p66 connection subdomain interact with the primer strand 11–15 nucleotides upstream of the primer 3′-terminus in all RT complexes with nucleic acid. Interactions of the p66 thumb with the nucleic acid involve helix αH of the p66 thumb, which is partly inserted into the minor groove of the dsDNA and helix αI, which is directly adjacent to the backbone of the template strand. Biochemical studies have shown that changes in residues of the αH, αI helices or in the DNA primer grip alter nucleic acid binding and may affect the polymerase and/or RNase H activities of RT [8,20,39–47].

**Figure 2.**
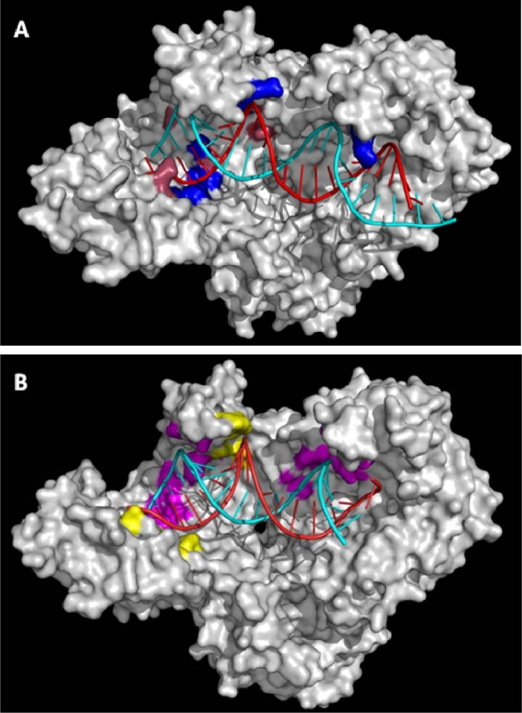
Interactions of RNA/DNA and DNA/DNA template/primers with HIV-1 RT. The two different template/primers (cyan/red) bind in the nucleic acid binding cleft of RT in a similar way. The RNA/DNA (panel A; PDB code 1hys) maintains the protein contacts seen in the complex with DNA/DNA (panel B, PDB code 2hmi) (these contacts are not shown in panel A. and has thirteen additional contacts. Nine of these contacts are through the 2′-OH group of the RNA sugar backbone (blue), whereas four are mediated through phosphate-backbone of RNA/DNA (plum). In panel B, the template-protein contacts are colored yellow and the primer-protein contacts are colored magenta.

The contacts between RT and the primer strand are very similar in RT structures with various DNA/DNA substrates. However, in the RT-RNA/DNA structure almost half of the RNA template residues have additional interactions with RT through their 2′-OH group (Figure 2). Finally, an RNA template has additional interactions with p51 residues at the floor of the nucleic acid binding cleft. The more extensive contacts between RT and RNA/DNA versus DNA/DNA may account for the increased polymerization activity and processivity of the enzyme with RNA templates.

### 4.2. dNTP Binding Site

**Figure 3.**
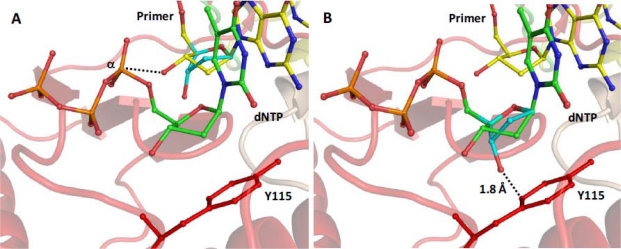
**A.** The effect of sugar ring conformation on catalysis of DNA synthesis by HIV-1 RT. Sugar-ring conformation of the nucleotide at the 3′-primer end (panel A). For efficient DNA synthesis to occur, the sugar ring conformation of the nucleotide at the 3′-primer end should be in the north (2′-exo/3′-endo) conformation (panel A, shown in yellow). The south (2′-endo/3′-exo) conformation (panel A, shown in cyan) of the sugar ring at the primer terminus mispositions the primer 3′-OH away for an in-line nucleophilic attack on the α-phosphate of the incoming dNTP (green), thereby resulting in inefficient catalysis. **B.** The sugar-ring conformation of the incoming dNTP should be north (panel B, green). If the incoming dNTP or nucleotide analog were to have a south conformation (panel B, cyan) this would result in steric hindrance with the aromatic ring of Y115 (shown in red). Thus, the favored conformation of the incoming dNTP is the north conformation (panel B, green). The software Coot [55] was used to prepare various sugar ring conformations of the primer terminus and incoming dNTP, starting from the structural coordinates of the HIV-1 RT/DNA/dNTP ternary complex (PDB Code 1rtd).

Comparison of the structures of binary (RT-DNA/DNA or RT-RNA/DNA) [8–13,20] and ternary (RT-DNA/DNA-dNTP or RT-DNA/DNA-TFV diphosphate) complexes [12,13,23] suggests that the overall conformation of the nucleic acid is maintained after a dNTP or an analog binds at the nucleotide binding site. The part of the template/primer that is close to the polymerase active site has bases in the C3’-endo (North) conformation (A-form), as is the case with DNA bound to other DNA polymerases [48–50]. This type of geometry allows optimal alignment of the primer 3′-OH for nucleophilic attack on the α–phosphate of the incoming dNTP (Figure 3A) [51]. Interactions of Y115 with the ribose ring of the incoming dNTP or its analogs, impose strict conformational requirements for substrate binding. Hence, only nucleotides with ribose bases in the North conformation can be accommodated at the dNTP-binding site (Figure 3B). Moreover, Y115 acts as a steric gate, hindering binding and incorporation of ribonucleotides [52,53] and affecting the processivity of the enzyme [54].

**Figure 4.**
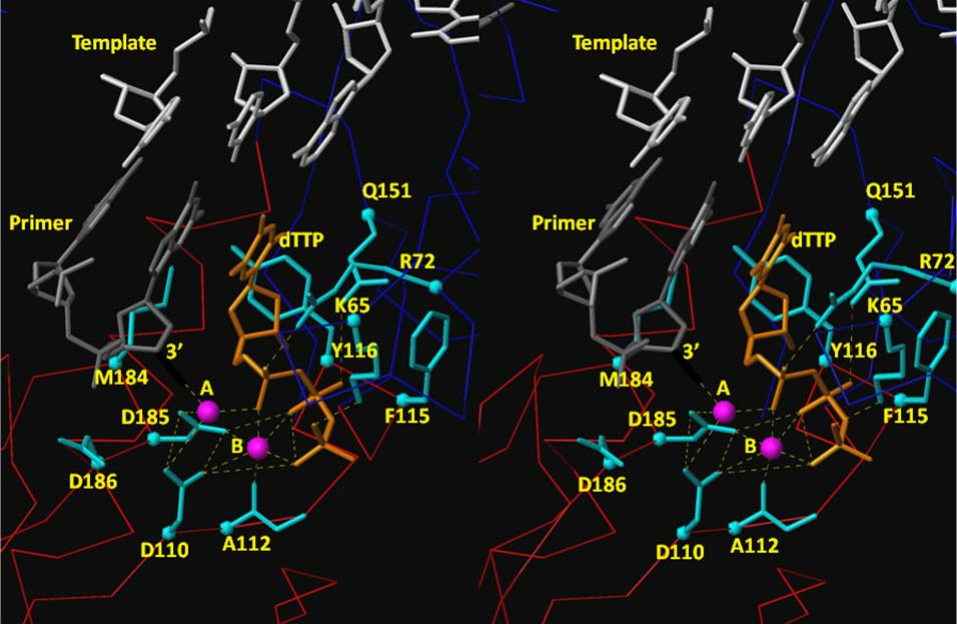
Stereo-view of the polymerase active site of HIV-1 RT. The primer strand is shown in gray, the incoming dNTP in orange, and the RT active-site residues interacting with substrates or metal ions in cyan. Metal ions A and B are shown as magenta bullets. The Cα-traces of the p66 palm and fingers subdomains are shown in red and blue, respectively. The yellow dotted lines depict the coordination geometry of the metal ions and the interactions of p66 fingers-subdomain residues with the incoming dNTP. The coordination geometry of metal ion B (also known as structural metal ion) is octahedral. Due to lack of the primer 3′OH group in the crystal structure (PDB file 1rtd), the coordination of metal ion A (known as catalytic metal) is incomplete. Interactions of fingers-subdomain residues K65, R72, and Q151 with dNTP are also shown in yellow dotted lines. Figures 4, 5, and 7 were generated by MolMol [58].

**Figure 5.**
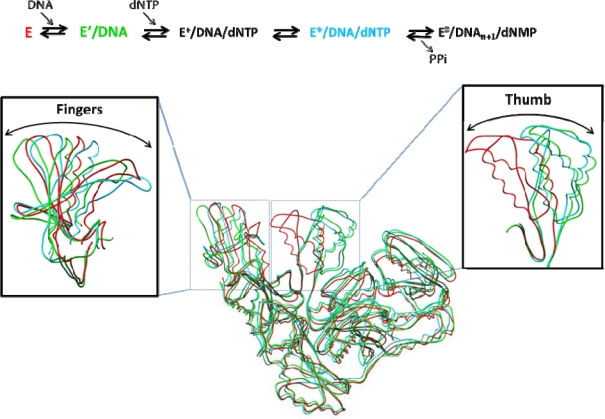
Conformational changes of p66 fingers and thumb subdomains during DNA synthesis by HIV-1 RT. Similar to other nucleic acid polymerases, HIV-1 RT undergoes conformational changes at various steps of the catalytic cycle. In the unliganded HIV-1 RT (E, shown as red tracing), the fingers and thumb subdomains fold over the active site to render it inaccessible. Binding of the template/primer opens up the fingers and thumb subdomains to accommodate the DNA/DNA or RNA/DNA substrates (green tracing). The binding of incoming dNTP causes the p66 fingers subdomain to move to a closed form and trap dNTP in a catalytically competent conformation (cyan tracing). After incorporation of dNMP, release of PPi, and translocation of the elongated template/primer, HIV-1 RT assumes the conformation seen in enzyme-DNA bound structure (green).

Three aspartates form the catalytic center of the RT polymerase active site, the 3′-OH group of the primer strand and the phosphate groups of the incoming dNTP, bind two divalent metal ions (Figure 4). They are part of a β-sheet composed of β-strands 7, 9, and 10. Two of the three aspartates, D185 and D186, belong to the conserved YMDD motif. Moreover, M184 also interacts with the 3′ primer terminus and the incoming dNTP and is also involved in NRTI drug resistance [10,56,57]. Binding of dNTP induces a major conformational change of the p66 fingers subdomain, which folds over the incoming nucleotide triphosphate (Figure 5). As a result, conserved residues R72 and K65 of the fingers bind the β- and γ-phosphates of the dNTP, respectively [12], completing the “engulfment” of the nucleotide by residues of the palm and thumb subdomains. The 3′-OH of dNTP interacts with residues of the 3′ ‘pocket’ [12]. This pocket is formed by conserved residues D113, A114, Y115, F116, and Q151 (Figure 4).

## 5. Structural Aspects of the Mechanism of DNA Synthesis by HIV-1 RT

The mechanism of DNA synthesis by HIV-1 RT has been studied extensively. Early crystal structures have aided to the characterization of the mechanism of DNA synthesis by providing structural insights and by guiding the design of biochemical experiments. The minimal kinetic mechanism of DNA polymerization by RT is similar to that established for other DNA polymerases [59–63] and it involves the following steps. (i) Binding of template/primer by RT. As mentioned earlier, binding of nucleic acid results in a large conformational change of the p66 thumb, moving it in an upright position to accommodate the nucleic acid (Figure 5); (ii) Initial binding of dNTP and metal to the RT-DNA complex to form a ternary complex. While there is no RT structure of this type, a related structure of a KlenTaq-DNA/DNA-dNTP complex [49] suggests that in the early stage of dNTP binding, the p66 fingers subdomain may be in an ‘open’ conformation; (iii) Conformational transition of the ternary complex to a catalytically competent ternary complex. This is considered to be the rate-limiting step, during which the p66 fingers fold down towards the RT catalytic residues, and the polymerase active site assumes a “closed” conformation (Figure 5). (iv) Formation of the phosphodiester bond between the α-phosphate of the nucleotide and the 3′-OH of the primer (chemical step), followed by (v) another conformational change, and (vi) release of pyrophosphate (PPi). Although there is no direct structural information in HIV-1 RT for the last two steps, the crystal structure of T7 RNA polymerase has demonstrated that the fingers subdomain adopts an ‘open’ conformation after PPi release [64]. Thus, it is likely that the release of PPi allows the p66 fingertips to regain their ‘open’ position, leading to binding of the next dNTP. (vii) Enzyme translocation along the DNA substrate. Direct comparison of pre- and post-translocation RT-DNA structures suggests that the conserved YMDD loop changes conformation during the translocation event, acting as a springboard that helps to propel the primer terminus after dNMP incorporation [11,13]. Biochemical data by Götte and colleagues demonstrate that RT can bind specific DNA sequences in a pre-translocation mode indicating that in some cases additional factors may be needed for efficient translocation of the elongated primer [65,66]. Thus, the exact mechanism of enzyme translocation to next template-position involves a conformational change or sequence context of template-primer or both remains a matter of discussion. Regardless of the mechanism of translocation, at this step the polymerase may either dissociate from the nucleic acid substrate (distributive mode) or continue synthesis (processive mode) (Figure 5). Based on structural data, a two divalent metal ion mechanism has been proposed for polymerase-catalyzed DNA synthesis [12,48–49,67–70]. One metal, also known as catalytic metal or metal A, coordinates with the 3′-OH of the primer strand and facilitates the nucleophilic attack on the α-phosphate of the incoming nucleotide; the other metal, known as structural metal or metal B, neutralizes the charge of the PPi leaving group. The model is supported by extensive structural data with DNA pol β, T7, and Taq DNA polymerases in complex with nucleic acid and dNTP [49] (Figure 4).

## 6. Structural Aspects of HIV-1 RT Inhibition Mechanisms

Due to its essential role in the viral life-cycle, HIV-1 RT has been a prominent target of anti-AIDS therapies. For this reason nearly half of approved anti-AIDS drugs inhibit the polymerase activity of RT. Inhibitors of HIV-1 RT belong to one of the two broad classes: the nucleoside RT inhibitors (NRTIs) and the nonnucleoside RT inhibitors (NNRTIs). While some specific inhibitors of the RNase H activity of RT have also been described, none has yet been approved for the treatment of HIV infections.

### 6.1. Nucleoside Reverse Transcriptase Inhibitors (NRTIs)

**Figure 6.**
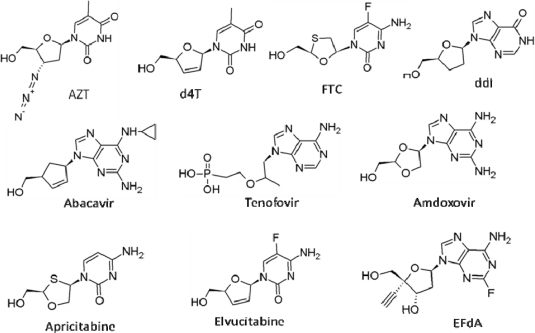
Nucleoside inhibitors (NRTIs) of HIV-1 RT: Chemical structures of NRTIs. With the exception of EFdA, all NRTIs lack a 3′ OH moiety. After their incorporation into DNA by HIV-1 RT, they act as chain terminators. In contrast, EFdA contains a 3′-OH and acts as a translcoation-defective reverse transcriptase inhibitor. FTC is 5-fluoro derivative of 3TC; the latter has not been included in this figure. Chemical structures were drawn with Chem Sketch 3.5 [82].

The first anti-AIDS drugs were 3′-OH-modified NRTIs aimed at exploiting the requirement of free 3′-OH by HIV RT. Currently, all approved NRTIs (Figure 6) lack a 3′-OH and act as chain terminators after their incorporation into viral DNA by RT. The potency of NRTIs is primarily affected by three major factors: (i) the efficiency by which they are converted to the active species, which is the triphosphate form [71,72]. Activation of NRTIs is carried out by cellular kinases that add the α-, β-, and γ- phosphates to the nucleoside prodrugs [73]. Tenofovir (TFV) already contains a phosphonate group and requires the addition of only two phosphates; (ii) NRTIs must be reasonably stable to catabolic enzymes present in the host cell environment, and (iii) their triphosphate form must be an efficient inhibitor of HIV RT. Notably, the absence of a 3′-OH from all approved NRTIs significantly decreases their activation efficiency, binding interactions with RT, and overall potency [74,75].

RT incorporates NRTI triphosphates with variable efficiencies. For example, RT incorporates zidovudine-5′-triphosphate (AZT-TP), a thymidine analog (Figure 6), very efficiently [76]. In contrast, lamivudine-5′-triphosphate (3TC-TP), an L-enantiomer analog of cytidine, is incorporated considerably less efficiently [77]. The crystal structures of RT bound to zidovudine-5′-monophosphate (AZT-MP)-terminated primer in pre- and post-translocation conformations revealed interactions of the 3′-azido of AZT-MP with residues of the active site (D113 and A112) that are likely to be responsible for the efficient binding of zidovudine (AZT) at the dNTP binding site [11]. On the other hand, modeling 3TC-TP instead of dTTP at the active site of the catalytic complex of RT-DNA/DNA-dTTP suggested that the L-enantiomer would be less favorable than the canonical substrates [10].

The crystal structures of the catalytic complex of RT with DNA/DNA and tenofovir diphosphate highlighted the role of K65 and R72 in binding the phosphonate and phosphate moieties of the incoming inhibitor [13,23]. In the same structure, Q151 and Y115 were shown to interact with the aliphatic (acyclic) component of TFV.

Recently, it was shown that 4′-ethynyl-2-fluoro-2′-deoxyadenosine (EFdA) (Figure 6), a nucleoside analog that unlike known chain-terminators retains a 3′-hydroxyl moiety, can inhibit HIV-1 RT with an unprecedented potency (EC_50_ = 50 pM in Peripheral Blood Mononuclear Cells), several orders of magnitude better than any known NRTI [78]. This exceptional antiviral activity is the result of multiple factors, including: a) an efficient phosphorylation of the prodrug due to the presence of a 3′OH; b) a resistance to catabolic degradation by adenosine deaminase [79], and c) a mechanism of action that is different from all other approved NRTIs. RT can use EFdA-5′-triphosphate (EFdA-TP) as substrate even more efficiently than the natural substrate dATP [79]. Surprisingly, despite the presence of a 3′-OH, the incorporated EFdA-5′-monophosphate (EFdA-MP) acts mainly as a *de facto* terminator of further RT-catalyzed DNA synthesis, due to difficulty of RT translocation on the nucleic acid primer possessing 3′-terminal EFdA-MP. Thus, EFdA is a Translocation Defective RT Inhibitor (TDRTI) that blocks HIV replication by a novel mechanism of inhibition [79].

### 6.2. Nonnucleoside Reverse Transcriptase Inhibitors (NNRTIs)

**Figure 7.**
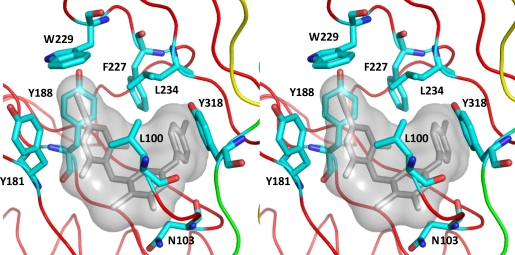
Stereo-view of the NNRTI binding site. NNRTIs bind in a pocket formed primarily by hydrophobic residues, which are rendered as cyan sticks with N and O atoms colored blue and red, respectively. A recently approved NNRTI, etravirine, is shown in gray. The p66 palm, thumb, and connection subdomains are shown in red, green, and yellow, respectively. Structural coordinates are from PDB code 1sv5.

NNRTIs are important components of several combination therapies. They bind in a hydrophobic pocket of HIV-1 RT, close to the polymerase active site and at the base of the p66 thumb. This pocket is formed by residues L100, K101, K103, V106, T107, V108, V179, Y181, Y188, V189, G190, F227, W229, L234, and Y318 of p66, and E138 of p51 [5,17] (Figure 7). Comparison of structures in the presence and absence of NNRTIs showed that the NNRTI-binding pocket (NNIBP) does not exist in the absence of NNRTIs [14,21,28,80]. Instead, it is created upon the binding of NNRTIs by large-scale conformational changes in the side chains of RT residues, including Y181 and Y188, and by moving the “primer grip” to an extended conformation [80]. NNRTI binding renders constraints upon the conformation of the p66 thumb such that it stays in an over-extended conformation [17,21]. It has been reported that some potent NNRTIs enhance the dimerization of HIV-1 RT [81]. There is no NNRTI binding pocket in p51 because the relative position of the RT subdomains is different than in p66.

**Figure 8.**
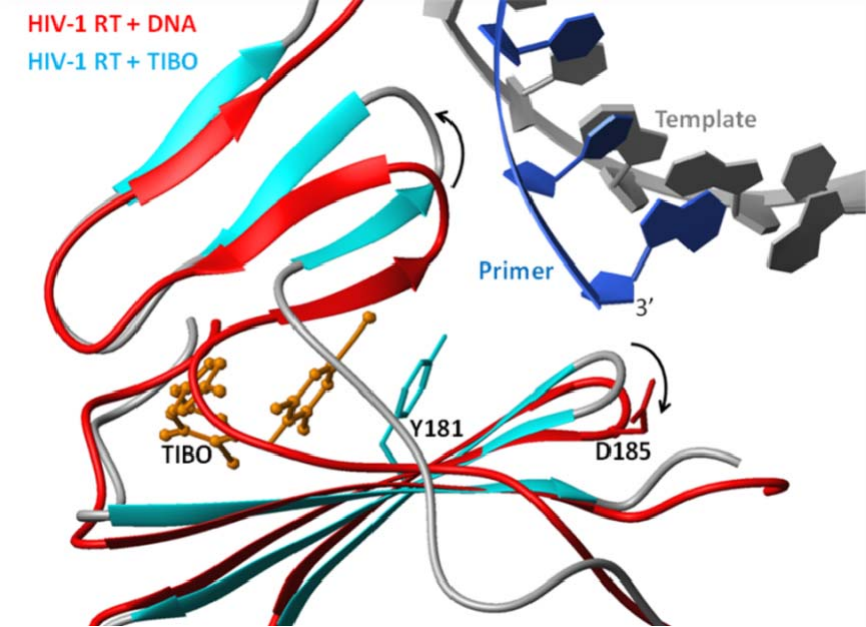
Mechanism of NNRTI inhibition: NNRTI-binding alters the geometry of the p66 thumb subdomain, changes the position of the ‘primer grip’, and causes misalignment of important components at the polymerase active site. The structure of RT in complex with TIBO (cyan and gray ribbons) is superposed onto the DNA-bound RT (red) to demonstrate the shift in the position of ‘primer grip’ as a result of NNRTI binding. Residues Y181 and D185 are shown as reference points for the NNRTI binding pocket and YMDD loop, respectively. The TIBO inhibitor is shown as orange balls and sticks, whereas the template/primer (gray/blue) is shown as ribbons and plates.

Biochemical data have shown that NNRTIs are non-competitive RT inhibitors with respect to either dNTP or nucleic acid substrates. Based on transient kinetics data, it has been proposed that binding of an NNRTI interferes with the chemical step of DNA synthesis [83,84]. A number of different mechanisms have been proposed for NNRTI inhibition of RT. Based on the early RT-nevirapine structure [17], it was postulated that nevirapine somehow alters the precise geometry of the polymerase active site, or restricts the mobility of the p66 thumb subdomain. The support for this hypothesis stems from the comparison of the RT-nevirapine structure with RT-DNA/DNA complex [20,80]. The comparison showed that NNRTI-binding causes local distortions of polymerase active site residues at the β9-β10 hairpin, as well as residues of the “primer grip” (β12-β13 hairpin) (Figure 8). These distortions may prevent proper positioning of the primer strand relative to the polymerase active site. Esnouf *et al*. reiterated that displacement of active site residues is likely to affect DNA synthesis [15]. Furthermore, Ding *et al*. [14,28] as well as Ren *et al*. [21] showed that other RT-NNRTI structures have striking similarity in the binding modes of diverse non-nucleoside inhibitors and remarkable consistency of a butterfly-like shape adopted by the inhibitor molecules suggesting the impact on polymerase activity by NNRTIs *via* a common mechanism. More recently, structural evidence was presented to demonstrate that the binding of NNRTIs restrict the flexibility of the YMDD loop and prevent the catalytic aspartate residues from adopting the metal-binding conformation seen in the RT-DNA/DNA-dTTP complex [12]. In summary, structural studies have shown that NNRTIs alter the geometry of the thumb, of the polymerase active site (YMDD motif and metal binding residues), and of the primer grip. Structural differences in the binding of various NNRTIs suggest that is possible that different NNRTIs have differences in their inhibition mechanism.

The above mechanisms of action of NNRTIs have been extrapolated from the comparison of apo RT, NNRTI-bound RT and ternary complex (containing nucleic acid and nucleotide) of RT. A key missing structure of RT complexed with template-primer, dNTP, and NNRTI would elucidate the structural changes that NNRTIs cause to the catalytic complex.

## 7. Molecular Mechanisms of Resistance

The emergence of HIV strains that are resistant to antiretrovirals is a consequence of incomplete virus suppression, high replication rates, and error prone DNA synthesis. There are known resistance mutations for all approved NRTIs. The availability of extensive structural information with wild-type and drug-resistant mutant RTs has helped to understand the molecular mechanisms of drug resistance to NRTIs and NNRTIs. These structures have also provided valuable insights toward designing novel inhibitors with improved resistance profiles. In this review we examine briefly the contribution of structural information in the elucidation of the mechanisms of RT resistance to NRTIs and NNRTIs.

### 7.1. Resistance to NRTIs

All approved NRTIs lack the 3′-OH found in the canonical dNTP substrates. Once incorporated, NRTIs act as chain terminators, blocking further DNA synthesis [71,72,85]. The NRTI-resistant RTs prevent NRTIs from blocking DNA synthesis, and are still able to incorporate normal dNTPs (or the virus would not be able to replicate). So far, HIV RT has used two main strategies for developing resistance to NRTIs (reviewed in [86] and references within):

### 7.2. Interference with the Incorporation of NRTIs

Residues that interfere with the incorporation of NRTIs reside in the p66 fingers or palm subdomains of RT; all are in positions that could affect the binding of an incoming dNTP or NRTI. A classic example of this mechanism is the high-level resistance to lamivudine (3TC) and emtricitabine (FTC), which is imparted by a single mutation at codon 184 of the RT polymerase active site (M184V or M184I) [87]. The M184V and M184I mutations also decrease viral replication capacity, particularly in the presence of low concentrations of dNTP [88,89]. In addition, M184V confers low resistance to zalcitabine (ddC), didanosine (ddI), and abacavir (ABC) [57,90–93]. Structural work has suggested that resistance to 3TC is caused by unfavorable (steric) interactions between β-branched amino acids (Val, Ile, Thr) and the β-L-oxathialone ring of 3TC triphosphate [10]. These interactions interfere less with the incorporation of dNTPs; thus the β-branched amino acids act as a molecular filter against 3TC [94]. Based on structural work, Sarafianos *et al*. proposed that NRTIs with a decreased footprint would have a more favorable profile against M184 mutants [11]. This prediction is confirmed by the favorable resistance profile of the FDA-approved acyclic drug TFV, and three more drugs currently in clinical trials (amdoxovir [95], elvucitabine [96,97] and apricitabine [98]) (Figure 6).

Other examples of mutations affecting the incorporation of NRTIs include the Q151M complex (Q151Mc: Q151M followed by A62V, V75I, F77L and F116Y), L74V, and K65R. The Q151M complex mutations cause multidrug resistance to AZT, ddI, ddC, stavudine (d4T), and ABC, but the mutant enzyme remains sensitive to adefovir-diphosphate (an acyclic NRTI related to tenofovir-diphosphate) and to 3TC-triphosphate [99–102]. Crystal structures of RT in complex with substrates [10–12,20,23] have provided insights into the molecular mechanism by which mutations at these residues alter the interactions of the incoming dNTP (or NRTI triphosphate) and cause resistance. The mechanism of Q151Mc multi-drug resistance involves a reduction of the catalytic rate (*k*_pol_) of NRTI incorporation, as compared to the canonical dNTP. The decrease in *k*_pol_ may be due to the loss of the hydrogen bond network involving the 3′-OH group of the incoming dNTP, residues Q151 and Y115, and the leaving PPi group [12,13,23,103].

The L74V mutation causes resistance to ddC, ddI [104,105], and ABC [92,93] by decreasing the incorporation rate of the inhibitor, as shown by steady-state kinetics studies [105]. The discrimination appears to be the effect of reduced catalytic rate (*k*_pol_) by L74V compared to WT enzyme [106]. Similar to Q151, L74 is important for the structural integrity of the dNTP-binding site, as it stabilizes the templating base opposite the incoming dNTP or NRTI substrates [9,12,20]. Changes in these interactions selectively reduce the incorporation rate of the nucleotide analogs and cause NRTI resistance [106]. Mutation at a neighboring residue (V75T) causes d4T resistance also by affecting the stability of the nucleotide-binding site through its proximity to residue 74 [107–109].

The K65 residue is located in the fingers subdomain of RT and interacts with the γ-phosphate of the incoming nucleotide substrate in the ternary complexes of HIV-1 RT [12]. This residue also interacts with the terminal phosphate of TFV-DP [13,23]. The K65R mutation has been found to confer resistance to TFV [110], but also to ddI and ABC, and to a lesser extent to 3TC and ddC [90,92,93,111]. Pre-steady state kinetic characterization of the K65R mutant suggested the discrimination against ddC-TP, ddA-TP, 3TC-TP and TFV-DP was due to either higher K_d_ or lower *k*_pol_ or combination of both [106,112–115]. In the case of TFV-DP, the discrimination by K65R results in a reduced *k*_pol_ [116,117] but for ddC-TP both K_d_ and *k*_pol_ are altered [114].The recent crystal structures of K65R RT with tenofovir diphosphate (TFV-DP) or dATP have shown that the guanidinium planes of the arginines K65R and R72 stack and restrict the conformational adaptability of both the residues, which explains the negative effects of the K65R mutation on nucleotide incorporation. Furthermore, the guanidinium planes of K65R and R72 are in two different rotameric conformations in TFV-DP and dATP-bound structures, which may help explain how K65R RT discriminates the drug from substrates [23].

### 7.3. Excision of Incorporated NRTIs

In the case of some NRTIs, the resistant enzymes readily accept the inhibitor as a substrate for incorporation into the DNA chain. However, these mutant RTs have an enhanced capacity for removing the chain-terminating nucleotide from the DNA terminus to generate a free 3′-OH and allow elongation of the formerly terminated DNA strand to resume [118,119]. The excision reaction is related to the normal DNA polymerization but in the reverse direction [118,119], and it requires a pyrophosphate donor which RT joins to the NRTI at the 3′ primer terminus, excising it from the primer DNA. Although both ATP or PPi can serve as pyrophosphate donors [118–122] it is now widely accepted that resistance is caused by the nucleophilic attack of ATP at the primer terminus to generate a free 3′-OH and a 5′-5′ dinucleoside-tetraphosphate [119]. Interestingly, the dinucleotide tetraphosphate product of ATP-mediated excision of chain-terminating AZT-MP is a potent chain-terminating substrate for HIV-1 RT [123].

AZT and d4T-resistance mutations M41L, D67N, K70R, L210W, T251Y/F and K219Q/E are the most common examples of excision enhancing mutations (EEM). Although not selected by TFV, these mutations also confer cross-resistance to this inhibitor. These residues are not located at the dNTP-binding site [12,13,23]. Biochemical data have shown that the presence of EEMs does not affect the incorporation of nucleotide analogs [124]. Instead, they facilitate the ATP-dependent removal of the incorporated NRTI that occurs at the same active site as the polymerization reaction [121].

**Figure 9.**
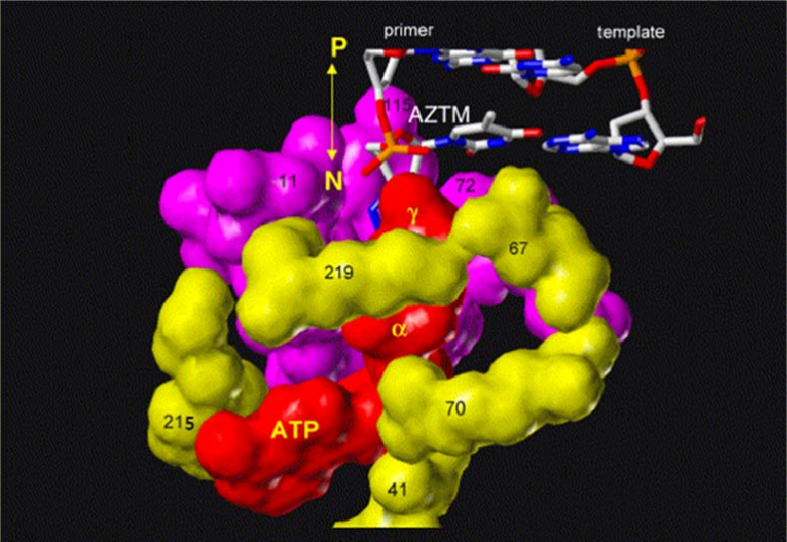
Structural components of NRTI resistance by the excision mechanism: Molecular model showing the binding of ATP (red) to AZT-resistant HIV-1 RT and unblocking of AZTMP-terminated primer. Van der Waals surfaces are drawn for polymerase active site residues (magenta) and residues involved in ATP binding and AZT resistance (yellow). Mutated amino acids M41L, K70R, L210W, and T215Y are shown with black labels. The two terminal nucleotide base pairs of the template/primer are shown. The 3′ end of the primer is AZTMP and is positioned at the N site. AZT-resistant enzyme has the amino acid substitutions M41L, D67N, K70R, T215Y, and K219Q. Y215 is likely to have aromatic interactions with the purine ring of ATP (red). R70 is likely to interact with the ribose ring and/or the α-phosphate of ATP. The wild-type amino acids at K219 and D67 were retained in the figure to show a potential salt bridge between the residues.

A structural model has been proposed to address the biochemical data of the excision reaction [116]. In this model, the ATP binding cleft is surrounded by residues involved in the excision-based resistance to NRTIs (41,44,67,70,210,215 and 219) (Figure 9). The aromatic ring of ATP interacts with the aromatic ring of Y215 through π-π interactions. This interaction changes the binding of ATP and affects its orientation. A recently presented crystal structure of WT and EEM-containing RTs complexed with a DNA/DNA primer/template and the ATP-dependent excision product of AZT (AZTppppA) confirmed this model [125].

For excision to occur, the chain-terminating nucleotide must be located in the nucleotide-binding site (the N-site) (Figure 9), in a pre-translocation conformation [11,126,127]. Structural and biochemical evidence have provided insights on the various factors that affect excision of NRTIs.

Excision of NRTIs is susceptible to the presence of the next incoming dNTP. This is because the presence of an incoming dNTP at the N-site stabilizes the NRTI-terminated primer to the post-translocation priming site (P-site) where it is not accessible for excision by nucleophilic attack by ATP [126,128]. If the NRTI is a dideoxy- inhibitor, then the resulting complex of RT with dideoxy-teminated DNA and incoming dNTP forms a stable “dead-end complex”, as shown in band mobility shift assays [129] and in the crystal structure of the corresponding complex [12] (Figure 4). However, the RT complex with AZT-MP terminated nucleic acid and incoming dNTP is not as stable [129]. Modeling of an incoming nucleotide in the crystal structure of the post-translocation complex of RT with AZT-terminated DNA (DNA _AZT-MP (P)_) predicts steric hindrance between the azido group of DNA _AZT-MP (P)_ and the incoming nucleotide. This suggests that the azido group prevents the next complementary nucleotide from inhibiting the excision reaction [11]. Primers terminated with a 3′ azido nucleotide (including AZT) have also been shown to bind preferentially in the pre-translocation state in the absence of dNTPs, properly positioning the chain-terminator for excision [128,130]. Binding of AZT-terminated primers in this mode appears to be enhanced by interactions of the azido group with RT residues of the N-site, as seen in the crystal structure of RT in complex with AZT-terminated DNA in pre-translocation mode [11].

The excision reaction is also affected by the presence of specific mutations. When added to RT with EEM mutations, the M184V mutation causes a decrease in AZT excision [131–133] and resensitization to AZT [134]. The decreased rate of excision in the presence of mutations at residue 184 may be related to a repositioning of the nucleic acid in the mutant enzyme, which may affect the alignment of the excision reaction components [10].

The K65R and L74V mutations [135–137], as well as the NNRTI resistance mutations L100I and Y181C [138–140], and the foscarnet resistance mutations W88G and E89K [141] also cause decreases in the excision reaction. While the structural details of this inhibition are not clear, some of the mutated residues (residues 74, 88, and 89) are in a position to affect the binding of the nucleic acid substrate and affect the alignment of the NRTI-terminated primer at the excision site.

The “fingers insertion complex” consists of an EEM mutation backbone with an additional T69S mutation and an insertion of two or more amino acids (usually SS, SA, or SG) between residues 69 and 70 of the fingers subdomain. This mutational pattern confers resistance to all NRTIs [142,143] through an increase in the rate of chain-terminator removal [144,145]. There is an apparent destabilization of the dNTP-induced dead-end complexes for primers terminated with a variety of NRTIs and an increase in their excision [146–148]. Site-specific foot-printing assays also showed that there was an increased access to the pre-translocational state [126] associated with a decreased binding for NRTIs and dNTPs [144].

Other RT mutations affect the specificity of the excision reaction in a different way. Such mutations are usually selected during HIV-1 treatment with multiple NRTIs. For example, while the excision enhancing mutations (EEM: M41L, D67N, K70R, L210W, T215Y/F, and K219Q) cause resistance to AZT, addition of the E44D/A mutation in the EEM background confers additional moderate resistance to 3TC [149]. It was shown that 3TC resistance due to mutation E44D/A is associated with the excision mechanism [150]. Such mutations are expected to affect the alignment of the ATP substrate of excision in the ATP-binding pocket (Figure 9).

Mutations at the connection subdomain of HIV-1 RT have been reported to affect drug resistance [151,152]. Specifically, N348I was reported to cause resistance to both the NRTI AZT, and the NNRTI nevirapine [153,154]. The AZT resistance mechanism appears to involve unblocking of AZT-terminated primers [155]. It has been proposed that due to their reduced RNase H activity, connection-domain mutant enzymes can excise incorporated AZT before the RNA template is degraded and the substrate becomes unusable by RT [155].

Wild-type HIV-2 RT has reduced ATP-dependent phosphorolytic activity in comparison with HIV-1 RT. When HIV-2 is challenged with AZT, it acquires resistance mutations that cause reduced AZT incorporation rather than enhanced excision of AZT-MP [156]. Comparison of the crystal structures of HIV-1 and HIV-2 RT [157] suggested that there are differences in the putative ATP binding sites of the two enzymes that explain why HIV-1 RT binds ATP more effectively and is more efficient in causing resistance to AZT through the excision mechanism [156].

## 8. Resistance to NNRTIs

**Figure 10.**
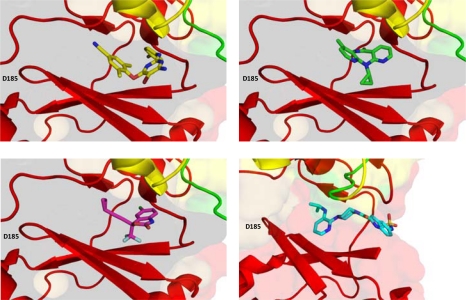
The NNRTI binding pocket (NNIBP) in various RT/NNRTI complexes. Clockwise from top left: RT/Etravirine (PDB Code 1sv5), RT/nevirapine (1vrt), RT/delavirdine (1klm), and RT/efavirenz (1ikw). Mutations conferring resistance to these drugs occur primarily in and around the NNIBP. Residue D185 of the YMDD loop is labeled as a polymerase active site reference point. The p66 palm, thumb, and connection subdomains are shown in red, green, and yellow, respectively.

Unlike the NRTI resistance mutations that are dispersed throughout the polymerase subdomain of RT, mutations conferring resistance to the four approved NNRTI drugs (etravirine, nevirapine, delavirdine, and efavirenz) are all located in, or around the NNIBP [158,159] (Figure 10). Commonly observed resistance mutations in HIV patients treated with NNRTI-based regimens include L100I, K103N, V106A, Y181C, Y188C/L, and G190A. These mutations occur alone or in combinations. Extensive structural studies of WT and NNRTI-resistant RTs in complex with NNRTIs have helped elucidate molecular mechanisms of NNRTI resistance [16,24,26,29,30,33]. Structural interpretations of the available clinical, cell culture and biochemical data have suggested that different sets of mutations cause drug resistance by at least three different general resistance mechanisms.

### 8.1. Loss/Change of Interactions at the NNRTI Binding Pocket

**Figure 11.**
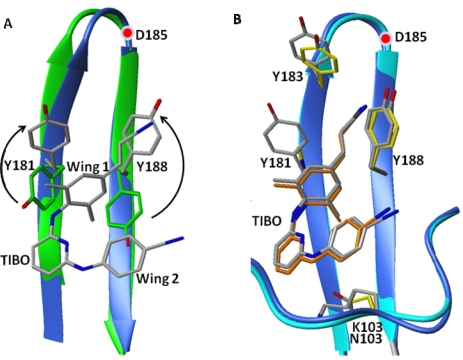
Binding of TMC-278 at the NNIBP results in a conformational change of residues Y181 and Y188. In panel A, comparison of unliganded RT (green ribbon and side chains) and RT/TMC-278 complex (blue ribbon and white side chains) shows that the aromatic rings of Y181 and Y188 “flip” as a result of TMC-278 binding to HIV-1 RT. Panel B shows the comparison of WT (blue ribbon, white side chains, and gray TMC-278) and Y181C/K103N (cyan ribbon, yellow side chains, and orange TMC-278) RT complexes with TMC-278. The yellow side chains belong to the TMC-278 bound K103N mutant RT. This figure also shows that the loss of interaction due to Y181C mutation is compensated by the interaction between cyanovinyl group and conserved Y183 (based on structures from pdb codes: 1dlo, 2zd1 and 3bgr).

Amino acids Y181 and Y188 are key residues of the NNIBP that interact with “Wing I” of the butterfly-like NNRTIs (Figure 11). Upon binding of nevirapine, loviride (α-APA), and tetrahydroimidazo[4,5,1-jkj][1,4]benzodiazepin-2(1H)-one (TIBO) [14,21,26,28,160] both tyrosines assume a “flipped” conformation (Figure 11) [154,156]. For weaker inhibitors such as 1-(2-2-hydroxyethoxymethyl)-6-(phenylthio)thymine (HEPT) and {3-[4-(2-methyl-imidazo[4,5-c]pyridin-1-yl)-benzyl]-3H-benzothiazol-2-one} (CP-94,707) only one chain is repositioned [31,161]. The large contribution of Y181 and Y188 to the binding energy of these NNRTIs explains why mutations at these residues result in a drastic decrease in inhibitor binding. Similarly, F227L also appears to cause resistance through loss of interactions with NNRTIs. Mutations L100I, V108A, and V18I have been proposed to cause minor changes, which in turn diminish the interactions of Y181 and Y188 with the bound NNRTI [33].

### 8.2. Steric Hindrance

The L100I mutation can cause steric interference between the β-branched isoleucine and an incoming NNRTI. Similarly, the G190A mutation can cause resistance through steric conflict of the alanine side chain and the bound NNRTI. For example, in the crystal structure of the WT RT/[(S)-4-isopropoxycarbonyl-6-methoxy-3-(methylthiomethyl)-3,4-dihydroquinoxaline-2(1H)-thione] (HBY097) complex [29] the bulky and rigid quinoxaline ring of HBY097 is near residue 190. A G190A mutation would cause the Cβ atom of A190 to have a steric clash with the quinoxaline moiety and reduce the binding of HBY097 [29].

### 8.3. Access to NNRTI binding pocket

Amino acids K101 and K103 are thought to be at the site of NNRTI entry to the pocket. Mutations K103N and K101E cause strong resistance to multiple NNRTIs, including the first generation NNRTI nevirapine. Comparison of the crystal structures of WT and K103N RTs in complexes with efavirenz [30], or HBY097 [29] demonstrated that the K103N mutation has minimal influence on the bound conformation of an NNRTI. However, in the crystal structure of K103N in the absence of NNRTI, a hydrogen bond between Tyr188 and Asn103 closes the entrance to the NNRTI binding pocket, efficiently reducing its access to multiple NNRTIs [16,24,30]. Moreover, this interaction results in a stabilized unbound state of RT and hence affects the kinetics of the inhibitor-binding process [16]. However, K103N has no significant resistance to etravirine, because this inhibitor was designed to interact with this residue, hence this mutation enhances its binding [161]. The kinetic of NNRTI binding have also been studied by the surface plasmon resonance technique showing that the resistance to NNRTIs may also be related to changes in the affinity for inhibitor either by reducing the association rate or by increasing the dissociation rate [162].

In summary, NNRTI-resistance mutations appear to affect NNRTI binding directly, by altering the size, shape, and polarity of different parts of the NNIBP or, indirectly, by affecting access to the pocket. To overcome the binding deficiency of NNRTIs to resistance mutants, more flexible NNRTIs were designed with so-called strategic flexibility [25, 163]. Flexible NNRTIs such as TMC-278 (rilpivirine) have compensatory interactions with RTs that have mutations causing resistance to the first-generation NNRTIs. TMC-278 uses its cyanovinyl group to recruit Y183 and help compensate for the loss of interactions caused by the Y181C mutation (Figure 11). This flexibility has been called “wiggling and jiggling” and allows NNRTIs to adapt to changes in the NNIBP caused by resistance mutations. Structural studies of WT, K103N/Y181C, and L100I/K103N HIV-1 RT complexes with TMC-278 [25] have shown that the side chains of the flexible NNIBP act as a molecular “shrink wrap” that makes a shape complementary to the optimized TMC-278 in WT and drug-resistant forms of HIV-1 RT.

## 9. Conclusions

Continuing advances in HAART appear to make a significant impact on life expectancy of HIV patients. However, the emergence of viral strains that are resistant to all known anti-AIDS drugs threatens the success of current antiviral therapies. Using structural studies to study the biochemical mechanisms of reverse transcription and to understand the mechanisms of inhibition and resistance to antiretrovirals has been a remarkably rewarding enterprise. To date, dozens of RT structures have been deposited in the Protein Data Bank (www.rcsb.org). These structures have contributed extraordinary details of the mechanism of viral replication, have inspired an enormous body of biochemical experiments that further expanded this knowledge, and have led to the design of new and improved therapeutics that are more potent against current multidrug resistance strains.
